# GnRH Agonist versus hCG Trigger in Ovulation Induction with Intrauterine Insemination: A Randomized Controlled Trial

**DOI:** 10.1155/2019/2487067

**Published:** 2019-03-13

**Authors:** Minh Tam Le, Dac Nguyen Nguyen, Jessica Zolton, Vu Quoc Huy Nguyen, Quang Vinh Truong, Ngoc Thanh Cao, Alan Decherney, Micah J. Hill

**Affiliations:** ^1^Department of OBGYN, Hue University of Medicine and Pharmacy, Hue University, 06 Ngo Quyen Street, Hue, Vietnam; ^2^Department of OBGYN, Walter Reed National Military Medical Center, Bethesda, MD, USA

## Abstract

This study is aimed at comparing clinical pregnancy rates (CPRs) in patients who are administered either gonadotropin-releasing hormone agonist (GnRHa) or human chorionic gonadotropin (hCG) for ovulation trigger in intrauterine insemination (IUI) cycles. A prospective randomized comparative study was conducted at Hue University Hospital in Vietnam. A total of 197 infertile women were randomly assigned to receive either GnRHa trigger (*n* = 98 cycles) or hCG trigger (*n* = 99 cycles) for ovulation trigger. Patients returned for ultrasound monitoring 24 hours after IUI to confirm ovulation. A clinical pregnancy was defined as the presence of gestational sac with fetal cardiac activity. There was no difference in ovulation rates in either group receiving GnRHa or hCG trigger for ovulation. Biochemical and CPR were higher in patients who received hCG (28.3% and 23.2%) versus GnRHa (14.3% and 13.3%) (*p* = 0.023, OR 0.42, 95%CI = 0.21 − 0.86 and *p* = 0.096, OR 0.51, 95%CI = 0.24 − 1.07, respectively). After adjusting for body mass index (BMI) and infertility duration, there was no difference in CPR between the two groups (OR 0.58, 95% CI 0.27-1.25, *p* = 0.163). In conclusion, the use of the GnRHa to trigger ovulation in patients undergoing ovulation induction may be considered in patients treated with IUI.

## 1. Introduction

Exogenous human chorionic gonadotropin (hCG) is commonly used to achieve final oocyte maturation and trigger ovulation in patients undergoing ovulation induction. In assisted reproductive cycles, however, hCG trigger is associated with a higher risk of developing ovarian hyperstimulation syndrome (OHSS) and premature luteinizing hormone (LH) surge [1]. It is widely accepted that the gonadotropin-releasing hormone agonist (GnRHa) can be used as an alternative with a comparative effect to hCG to achieve final oocyte maturation by inducing a LH and follicle-stimulating hormone (FSH) surge but decrease the risk of OHSS in in vitro fertilization (IVF) [2–5].

In patients undergoing ovulation induction with gonadotropins and intrauterine insemination (IUI), because the number of developed follicles is limited, routinely not over 3, the risk of OHSS is negligible. A potential benefit to employing the use of GnRHa trigger for IUI cycles may be to induce a more physiologic type of gonadotropin surge involving the flare effect of FSH and LH from the pituitary [5]. In the natural menstrual cycle, there is a midcycle surge of both gonadotropins [6]. The FSH peak contributes to the resumption of meiosis and cumulus expansion and induces LH receptors in the granulosa cells [7–10]. hCG trigger is used as a surrogate to mimic the LH surge and does not result in a release of FSH [11]. A study of IVF patients who were given a bolus of FSH in addition to the hCG trigger found better oocyte recovery and fertilization rates in comparison to hCG trigger alone [12].

Conversely, GnRHa-triggered cycles result in a shorter duration of LH release in comparison to the natural menstrual cycle. The corpus luteum, which is stimulated by LH, may be defective. Studies have shown a shorter duration of the luteal phase after GnRHa trigger [13]. It has been reported that GnRHa are associated with lower pregnancy rates in comparison to hCG for ovulation triggering [14]. In recent years, many studies have concluded that luteal phase support in IVF cycles triggered with GnRHa improves pregnancy rates while also significantly decreasing the risk of OHSS [14, 15]. One strategy to overcome potential luteal phase deficiency is to administer a low dose of hCG 35 hours to 5 days after GnRHa [16]. Intramuscular progesterone and transdermal estradiol (E_2_) are frequently advised to compensate for the defective corpus luteum [17].

Intrauterine insemination (IUI) combined with ovulation triggering is commonly the first choice of treatment for infertility due to its relative affordability and ease in comparison to IVF. The pregnancy rate in IUI cycles ranges from 7.5% to 20% [18–20]. The addition of controlled ovarian hyperstimulation, especially by exogenous gonadotropins, significantly improves the pregnancy rate [21]. While hCG is most commonly used to trigger ovulation in OI and IUI cycles, our study sought to compare the pregnancy rates of patients who were triggered with GnRHa versus hCG in patients undergoing either natural cycle or controlled ovarian stimulation with gonadotropins and IUI.

## 2. Materials and Methods

### 2.1. Study Design

A prospective randomized comparative study was conducted at Hue University Hospital in Vietnam from April 2016 to June 2017 in 197 infertile women undergoing IUI. The study was approved by the Ethics Committee at Hue University of Medicine and Pharmacy. The clinical study registration number is NCT03825445

### 2.2. Study Population

A total of 217 women were recruited into the sample at the first stage. Inclusion criteria were women with bilateral tubal patency, at least one follicle ≥ 18 mm in diameter on the day of trigger and men with more than five millions total motile sperm after preparation. Only the first cycles of IUI were studied, and 197 infertile women who obtained at least 1 mature follicle at the first cycle were included in analysis. Patients were randomly assigned to receive either GnRHa trigger (*n* = 98 cycles) or hCG trigger (*n* = 99 cycles) for ovulation trigger.

### 2.3. Intervention

All patients included in the study were subjected to complete history and physical examination. Patients with a history of abnormal menstrual cycles (amenorrhea, oligomenorrhea) underwent ovarian stimulation. Stimulation was started on cycle day eight with 75 IU Menogon (Ferring Pharm Co., Switzerland) daily. Ultrasound monitoring was required after every 2-3 days of stimulation, and adjustments to dose and duration were tailed according to the patient's response. Ovulation was triggered when at least one and no more than 3 follicles reached ≥18 mm in diameter. Patients were then randomly assigned to receive either two doses of GnRHa (Fertipeptil 0.1 mg × 2 vials; Ferring Pharm Co., Switzerland) or hCG (Pregnyl 5000 IU; Organon Pharm Co., Netherlands) for ovulation trigger.

IUI was then performed with sperm preparation by radiant centrifugation 36 hours after the trigger. Luteal phase support with progesterone 200 mg daily (Utrogestan; Besins Healthcare Co., Belgium) was started in the day of IUI.

### 2.4. Assessment of Outcomes

Patients returned for ultrasound monitoring 24 hours after IUI to confirm ovulation which is determined by the accumulation of free fluid in the peritoneum at Douglas sac and disappearance of the previous mature follicles.

Serum *β*-human chorionic gonadotropin (*β*hCG) was collected 14 days after insemination. A biochemical pregnancy was defined by *β*hCG concentration > 25 mIU/ml (Shapphire 350; Cork Co., Ireland). Two weeks after a positive *β*hCG test, the patient returned for an ultrasound appointment. A clinical pregnancy was defined as the presence of gestational sac with fetal cardiac activity.

### 2.5. Statistical Analysis

Statistical analyses were performed using the SPSS for the Windows version 19.0 program (SPSS Inc., Chicago, IL). All values are given as median, mean ± standard deviation (SD), and the statistical tests of chi-squared and one-way analysis of variance (ANOVA). A *p* value <0.05 was considered as statistical significance in other analyses.

## 3. Results

A total of 197 IUI cycles were included in analysis. Patients were randomly assigned to receive either GnRHa trigger (*n* = 98 cycles) or hCG trigger (*n* = 99 cycles) for ovulation trigger.

Table 1 shows no significant difference regarding age, infertility diagnosis, type, or duration, body mass index (BMI), and baseline fertility labs.

There was no difference in ovulation rates in either group receiving GnRHa or hCG trigger for ovulation. A significant increase in biochemical pregnancy rates (BPR) was found in natural IUI cycles triggered with hCG versus GnRHa (29.6% vs. 14.1%, *p* = 0.031). hMG-stimulated cycles did not exhibit a difference in BPR triggered with GnRHa or hCG. However, the overall BPR were greater in cycles triggered with hCG versus GnRHa (28.3% vs. 14.3%, *p* = 0.023). The CPRs were not significantly different for patients undergoing spontaneous or hMG-stimulated cycles in cycles triggered with GnRHa versus hCG. Analysis of all cycles (stimulated and spontaneous) did result in the increase in CPR in cycles triggered with hCG versus GnRHa but not significantly (23.2% vs. 13.3%, *p* = 0.096).

Cycles were analyzed by age, BMI, infertility duration, and ovulation stimulation to determine if the ovulation rate in GnRHa- or hCG-triggered cycles was influenced by these confounders (Table 2). In GnRHa-triggered cycles, women with a BMI less than 23 were more likely to ovulate (OR 0.13, 95% CI 0.02-0.76). In hCG-triggered cycles, patients with a history of infertility less than 2 years were also more likely to ovulate (OR 6.17, 95% CI 1.24-30.78). Age, infertility diagnosis, ovarian stimulation, and total days of hMG treatment did not influence ovulation rates.

After adjusting for BMI and infertility duration, there was no difference in ovulation rates (OR 0.56, 95% CI 0.23-1.38). In GnRHa-triggered cycles, patients were less likely to achieve a biochemical pregnancy than hCG-triggered cycles (OR 0.47, 95% CI 0.23-0.98). However, CPR was not lower in GnRHa-triggered cycles in comparison to hCG-triggered cycles (OR 0.58, 95% CI 0.27-1.25).

## 4. Discussion

In this study, BPR was increased in spontaneous cycles triggered with hCG vs. GnRHa (28.3% vs. 14.3%, *p* = 0.023). However, the CPR was not significantly different in spontaneous cycles in regard to the trigger used. There was a difference in CPR when both the spontaneous and stimulated cycles were included but not significantly; the CPR was lower in cycles triggered with GnRHa vs. hCG (13.3% vs. 23.2%, *p* = 0.096). After adjusting for BMI and infertility duration, the CPR between GnRHa and hCG cycles was not significant. Our findings are supported by a randomized controlled trial of 110 infertile women which found no difference in pregnancy rates in women who received a GnRHa or hCG trigger (26.9% vs. 20.8%, *p* = 0.46) [22].

Ovulation triggered by GnRHa is known to negatively impact luteal function and endometrial receptivity [13]. Ovulation induced with GnRHa results in the release of endogenous LH, which has a significantly shorter half-life than hCG, 60 minutes versus 24 hours [23]. The prolonged half-life of hCG is responsible for sustained release of vasoactive substances that increase vascular permeability and thus increases the risk of OHSS [24]. The shorter half-life of the GnRHa decreases the risk of OHSS as stimulation to the corpora lutea is reduced as reflected by lower levels of E_2_ and progesterone in comparison to hCG trigger [1].

The duration of the LH surge after GnRHa is shorter than the physiological surge of LH during the natural menstrual cycle. This predisposes cycles treated with GnRHa to luteal phase deficiency. The spontaneous LH surge is defined by a short ascending phase (14 hours), a peak phase (14 hours), and a descending phase (20 hours) while the GnRHa induced LH surge consisted of two phases: a short ascending limb (>4 hours) and a long descending limb (>20 hours) [25, 26]. The shortened surge may be responsible for accelerated degradation of the corpus luteum and a decrease in progesterone and E_2_ in cycles triggered by GnRHa versus hCG [25, 27]. While serum E_2_ and progesterone were not measured after trigger in our study, it can be hypothesized that their E_2_ and progesterone in the GnRHa cycles were decreased and may have negatively impacted BPR and CPR. It is important to note that our patients did receive progesterone 200 mg per day for luteal support. However, it is unknown if our luteal phase support was adequate, as no RCT has been conducted to determine the superior luteal phase support in cycles triggered with GnRHa. A meta-analysis, which found progesterone luteal support to be beneficial in patients undergoing ovulation induction with gonadotropins in IUI cycles, did recommend vaginal progesterone due to low cost and side effect profile. However, all included studies were triggered with hCG [28]. As progesterone is secreted in a pulsatile manner, there is no standard to define what value of progesterone results in luteal phase deficiency [29]. Significantly lower implantation rates, CPR, and a higher rate of early pregnancy loss have been documented in antagonist IVF cycles triggered with GnRHa versus hCG despite luteal support with oral E_2_ and vaginal progesterone [30]. A randomized controlled trial (RCT) did report similar ongoing pregnancy rates to hCG trigger when patients who were administered GnRHa trigger received low-dose hCG at the time of oocyte retrieval in addition to oral E_2_ and intramuscular progesterone [31]. Therefore, our patients may not have received adequate luteal support as only 200 mg of progesterone was prescribed daily.

Concerning the characteristics and outcome of the ovulation rate in GnRHa- and hCG-triggered cycles as seen in Table 2, in GnRHa-triggered cycles, women with a BMI less than 23 were more likely to ovulate (OR 0.13, 95% CI 0.02-0.76). In hCG-triggered cycles, patients with a history of infertility less than 2 years were also more likely to ovulate (OR 6.17, 95% CI 1.24-30.78). Age, infertility diagnosis, ovarian stimulation, and total days of hMG treatment did not influence ovulation rates. In a recent randomized clinical trial, Taheripanah et al. have compared the effect of GnRHa and hCG on final oocytes for ovulation triggering after stimulation with combination of clomiphene citrate and recombinant FSH; they found no differences in pregnancy, implantation, and fertilization rates between the two triggering agents, although they did not mention about the ovulation rate [22]. Similarly, in a study on intrauterine insemination timing choice reported by Yumusak et al., after ovulation induction of 280 infertile patients by using clomiphene citrate and trigger by hCG, they found no significant difference with respect to female age, duration of infertility, endometrial thickness, and number of mature follicles affecting the outcome among the groups [32]. This information has approved the fact that both GnRHa and hCG are effective in triggering ovulation, irrespective of the mentioned factors.

After adjusting for BMI and infertility duration, there was no difference in ovulation rates (OR 0.56, 95% CI 0.23-1.38) as shown in Table 3. Although in GnRHa-triggered cycles, patients were less likely to achieve a biochemical pregnancy than hCG-triggered cycles (OR 0.47, 95% CI 0.23-0.98). However, CPR was not lower in GnRHa-triggered cycles in comparison to hCG-triggered cycles (OR 0.58, 95% CI 0.27-1.25). By this result, it can be concluded that the use of the GnRHa to trigger ovulation in patients undergoing ovulation induction can be considered in patients treated with IUI.

One weakness of our study is the inclusion of both gonadotropin-stimulated cycles and natural cycles. We did not define the number of patients who underwent hMG stimulation or natural cycle. However, the ovulation rate did not significantly differ in hCG- or GnRHa-triggered cycles. Furthermore, because serum progesterone levels were not measured in the luteal phase, 200 mg vaginal progesterone supplemented per day as routine was not proven to be adequate to support the luteal phase. This might be the likely explanation for the lower pregnancy rate in the GnRH agonist group although the significant difference was not obtained.

In conclusion, our results suggest that GnRHa can be used to trigger ovulation in stimulated and natural cycles with IUI. Further, randomized clinical studies are needed to determine the live birth rates with GnRHa trigger versus hCG trigger in IUI cycles.

## Figures and Tables

**Table 1:** Demographic characteristics of the patient population and intervention outcomes between two groups.

Variables	GnRHa-triggered cycles (*n* = 98)	hCG-triggered cycles (*n* = 99)	Total
Mean age (years)	31.47 ± 5.34	31.30 ± 4.77	31.39 ± 5.05
Infertility duration (years)	2.74 ± 1.62	2.37 ± 1.83	2.55 ± 1.74
BMI (kg/m^2^)	20.10 ± 2.23	20.28 ± 2.77	20.19 ± 2.51
Infertility type			
Primary	65.3%	68.7%	67.0%
Secondary	34.7%	31.3%	33.0%
Infertility causes			
Male factor	80.6%	82.8%	81.7%
Polycystic ovary syndrome	34.7%	33.3%	34.0%
Decreased ovarian reserve	18.4%	10.1%	14.2%
Tubal factor	5.1%	5.1%	5.1%
Adenomyosis	4.1%	5.1%	4.6%
Basal hormonal level			
FSH (IU/L)	6.97 ± 2.47	7.42 ± 4.12	7.19 ± 3.40
LH (IU/L)	6.43 ± 4.17	7.26 ± 5.15	6.84 ± 4.70
AMH (ng/mL)	5.53 ± 6.36	6.95 ± 6.69	6.08 ± 6.49
Ovulation rate			*p* value
Spontaneous cycles	87.3%	91.4%	0.441
hMG stimulation	81.5%	83.3%	0.600
Total	85.7%	89.9%	0.392
Pregnancy rate			
Biochemical pregnancy rate			
Spontaneous cycles	14.1%	29.6%	0.031
hMG stimulation	14.8%	22.2%	0.694
Total	14.3%	28.3%	0.023
Clinical pregnancy rate			
Spontaneous cycles	14.1%	24.7%	0.110
hMG stimulation	11.1%	16.7%	0.670
Total	13.3%	23.2%	0.096

GnRHa: gonadotropin receptor hormone agonist; hCG: human chorionic gonadotropin; BMI: body mass index; FSH: follicle-stimulating hormone; LH: luteinizing hormone; AMH: anti-Mullerian hormone; hMG: human menopausal gonadotropins.

**Table 2:** Characteristics and outcome of the ovulation rate in GnRHa- and hCG- triggered cycles.

Variable		Ovulation rate in GnRHa-triggered cycles		Ovulation rate in hCG triggered-cycles	
		OR	95% CI/*p*	OR	95% (CI)/*p*
Age (years)	<35	0.78	0.22–2.76/0.741	1.31	0.26–6.65/0.546
	≥35				

BMI (kg/m^2^)	<23	0.13	0.02–0.76/0.036	0.65	0.07–6.03/0.537
	≥23				

Infertility diagnosis	Primary	0.95	0.29–3.10/0.578	4.58	0.55–37.83/0.165
	Secondary				

Infertility duration (years)	<2	2.57	0.79–8.31/0.181	6.17	1.24–30.78/0.019
	≥2				

Ovarian stimulation	Yes	0.64	0.19–2.11/0.522	0.47	0.11–2.04/0.383
	No				

Total duration of hMG (days)	≤3	0.44	0.06–3.42/0.580	2.29	0.17–30.96/0.500
	>3				

GnRHa: gonadotropin receptor hormone agonist; hCG: human chorionic gonadotropin; BMI: body mass index; hMG = human menopausal gonadotropins.

**Table 3:** Intervention outcomes after adjustment for BMI and infertility duration in GnRHa- and hCG-triggered cycles.

Outcomes	GnRHa-triggered cycles (*n* = 98)	hCG-triggered cycles (*n* = 99)	*p* value
Ovulation rate	OR 0.56 CI: 95% (0.23–1.38)		0.207
Biochemical pregnancy rate	OR 0.47 CI: 95% (0.23–0.98)		0.044
Clinical pregnancy rate	OR 0.58 CI: 95% (0.27–1.25)		0.163

GnRHa: gonadotropin receptor hormone agonist; hCG: human chorionic gonadotropin.

## Data Availability

The data used to support the findings of this study are available from the corresponding author upon request.
